# DEM modeling direct shearing behavior of sand considering anti-rotation of particle

**DOI:** 10.1038/s41598-023-34933-y

**Published:** 2023-06-02

**Authors:** Liming Wu, Jiangu Qian, Liangfu Xie, Yongjun Qin

**Affiliations:** 1 College of Civil Engineering and Architecture, Xinjiang University, Urumqi, 830046 China; 2 Department of Geotechnical Engineering, Tongji University, Shanghai, 200092 China; 3 Xinjiang Civil Engineering Technology Research Center, Urumqi, 830046 China; 4 Xinjiang Academy of Architectural Science (Limited Liability Company), Urumqi, 830054 China

**Keywords:** Civil engineering, Petrology

## Abstract

Considering anti-rotation of sand particles, two-dimensional Discrete Element Method (DEM) has been employed to reproduce direct shear behaviors of sand with different particle distribution sizes, so as to explore effects of anti-rotation of particle on responses of stress-displacement and dilatancy, the evolution law of shear stress, coordination number and vertical displacement of sand samples, and analyze the contact force chain, contact fabric and porosity of the samples after shearing.The results show that the anti-rotation ability of sand is enhanced, the torque of overcoming the relative rotation between particles is increased, and the peak shear stress, dilatancy and porosity in the middle of the sample are increased; with the increase of the anti-rotation coefficient, the coordination number decreases more obviously. The proportion of the contact number in the direction of 100°–160° to the total contact number decreases with the increase of the anti-rotation coefficient. The elliptical shape of the contact configuration becomes more flat, and the anisotropy of the contact force chain is more obvious; compared with fine sand, the coarse sand has greater shear capacity, more obvious dilatancy and larger porosity in the middle of the sample.The maximum minimum particle size ratio of the sample becomes larger, so that the shear strength of the sample is reduced, and the dilatancy is also weak.

## Introduction

Sand is a typical granular material.Sand’s particle shape, size and other properties affect the mechanical properties of it^1–3^. At present, there are many test methods to study the mechanical properties of sand, and the direct shear test is a classical and simple method to study the mechanical properties of sand^4–8^. Nowadays, a large number of scholars use discrete element method to simulate rock blasting, slope^9–11^, debris flow^12–14^, sand^15–17^ and so on. For sand direct shear, researchers use discrete element method to carry out numerical shear tests to study and analyze the number of particle contact, contact force, porosity, contact fabric, shear band size and other microscopic properties^18–20^. At present, there are many studies on direct shear of sand. Lu et al.^3^ used discrete element method to carry out direct shear test. Considering the shape of sand particles, it is found that the influence of particle shape on the mechanical properties of sand cannot be ignored. Zhou et al.^21^ conducted a series of direct shear tests by generating block samples with real particle shape and corresponding spherical samples, and found that the real particle shape greatly increased the interlocking of particles, thus revealing the microscopic mechanism of shape effect on particle interlocking. For the study of rolling friction contact model, Jiang et al.^22,23^ proposed a new two-dimensional and three-dimensional discrete model of granular materials with rolling resistance. The prediction results of the new model may be closer to the experimental observations. Antonio et al.^24^ used a rolling friction contact model to build a real sand shape model for direct shear tests.

It can be seen from the above that the researchers have done a lot of research on the direct shear test of sand. However, due to the extremely irregular shape and great randomness of sand particles, it is extremely inconvenient to consider the shape of sand particles, and the requirements for computer computing power are very high. When there are more particles and more particle shape types, the simulation test cannot be carried out efficiently^25^. The anti-rotation linear contact model can make the ideal spherical particles appear as granular materials composed of real particles to a certain extent^22^. Therefore, this paper uses the discrete element software PFC^2D^ (Particle Flow Code) to carry out direct shear tests on sand samples of different particle sizes. Under the vertical pressure of 150 kPa, the anti-rotation ability of the particles is analyzed and studied on the stress–strain relationship and the dilatancy of the samples in the direct shear process. The coordination number, contact force chain, contact fabric and porosity distribution are analyzed.

## Construction of discrete element model

### Anti-rotation model of discrete element

In this paper, the discrete element software PFC^2D^ is used to simulate the direct shear test. In the discrete element model, the motion characteristics of a single particle obey Newton 's second law:

1 $$ F_{i} = m_{i} \frac{{d^{2} }}{{dt^{2} }}x_{i} $$

2 $$ M_{i} = J_{i} \frac{d}{dt}\omega_{i} $$

$F_{i}$ is the resultant force acting on the particles; $m_{i}$ is particle mass; $x_{i}$ is the position of the particles; $M_{i}$ is the resultant moment of particles; $J_{i}$ is rotational inertia; $\omega_{i}$ is the angular velocity of particles.

The contact model between the particles of the sample is based on the anti-rotation linear contact model. A linear contact model is used between the particles and the wall. The anti-rotation linear contact model increases the anti-rotation torque based on the linear model^3,22,23^. It is defined as:

3 $$ M^{r} = \left\{ {\begin{array}{*{20}c} { - k_{r} \Delta \theta_{b} ,k_{r} \Delta \theta_{b} \le \mu_{r} \overline{R} F_{n}^{r} } \\ {\mu_{r} \overline{R} F_{n}^{r} ,k_{r} \Delta \theta_{b} > \mu_{r} \overline{R} F_{n}^{r} } \\ \end{array} } \right. $$

where $\mu_{r}$ is the anti-rotation coefficient; $\Delta \theta_{b}$ is the relative rotation between contact particles; $\overline{R}$ is the effective contact radius; $k_{r}$ is the rotational stiffness of particles; $F_{n}^{r}$ is the normal contact force.

### Direct shear model establishment and parameter setting

**Figure 1:**
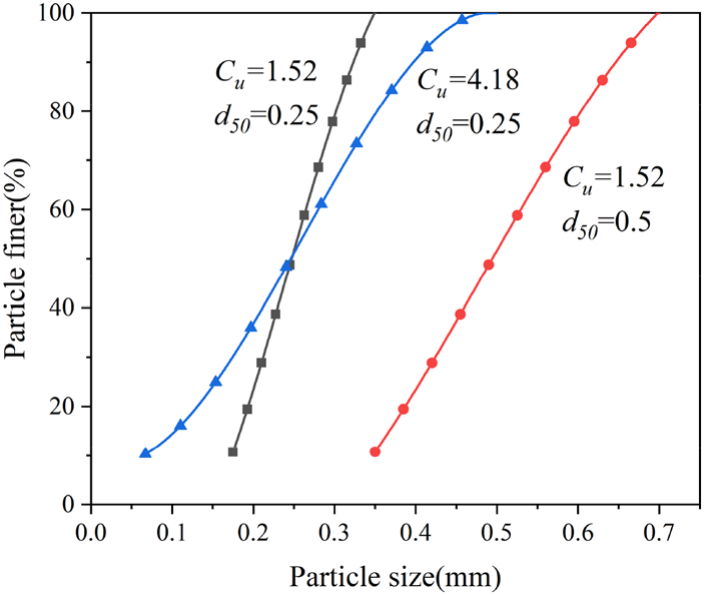
Particle size distribution.

**Figure 2:**
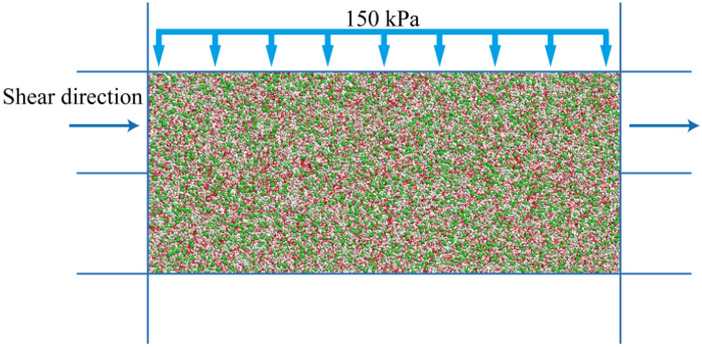
Direct shear test diagram.

**Figure 3:**
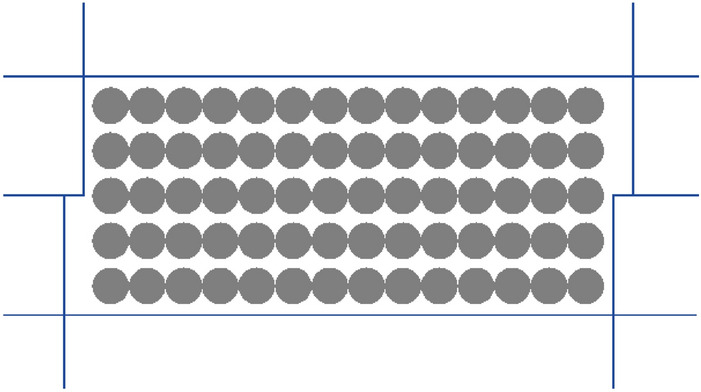
Measurement circle distribution.

The size of the sand discrete element direct shear specimen is set to 45 mm × 20 mm, and the specimen is randomly generated in the area surrounded by 8 walls. According to previous studies, in order to avoid the distortion of simulation results, the friction coefficient is 0.5. When the number of particles is greater than 2000 or the ratio of the minimum particle size to the sample length is less than 0.01, the model stability is better^3,24,26,27^. Therefore, with reference to previous studies, the main meso-parameters of the sample are shown in Table 1. In this paper, three kinds of sand with different particle sizes were used for direct shear numerical simulation test. The number of particles is shown in Table 2. The particle size range is shown in Fig. 1. The anti-rotation coefficient μ_r_ of the sample is 0.0, 0.1, 0.2, 0.3, 0.4. After the sample is formed, the confining pressure is applied to the sample by servo, and the confining pressure is 150 kPa. After the confining pressure is stable, the upper part of the sample is sheared at a constant speed of 0.005 m/s under the condition of maintaining the load stability in the vertical direction, and the test is completed when the shear displacement reaches 2 mm (Fig. 2). Measuring circles are arranged in the specimen to monitor porosity and contact fabric in the specimen (Fig. 3).

**Table 1:** Meso-parameters of sand.

Normal contact stiffness (Pa)	Shear contact stiffness (Pa)	Particle density (kg/m^3^)	Damping ratio	Ball friction coefficient	Wall friction coefficient	Initial porosity (%)	Anti-rotation coefficient
1.5 × 10^8^	1.0 × 10^8^	2670	0.7	0.5	0	15	0.0, 0.1, 0.2, 0.3, 0.4

**Table 2:** Particle size range and particle number of sand.

Category	Particle size range/mm	Dmax/Dmin	Coefficient of uniformity	Particle number
Sample 1 (fine sand)	0.175 ~ 0.35	2	*C*_u_ = 1.52 *d*_50_ = 0.25	15,561
Sample 2 (coarse sand)	0.35 ~ 0.7	2	*C*_u_ = 1.52 d50 = 0.5	3865
Sample 3	0.067 ~ 0.5	7.5	*C*_u_ = 4.18 *d*_50_ = 0.25	22,789

## Analysis of macroscopic mechanical properties

### Shear stress-displacemnt responses

**Figure 4:**
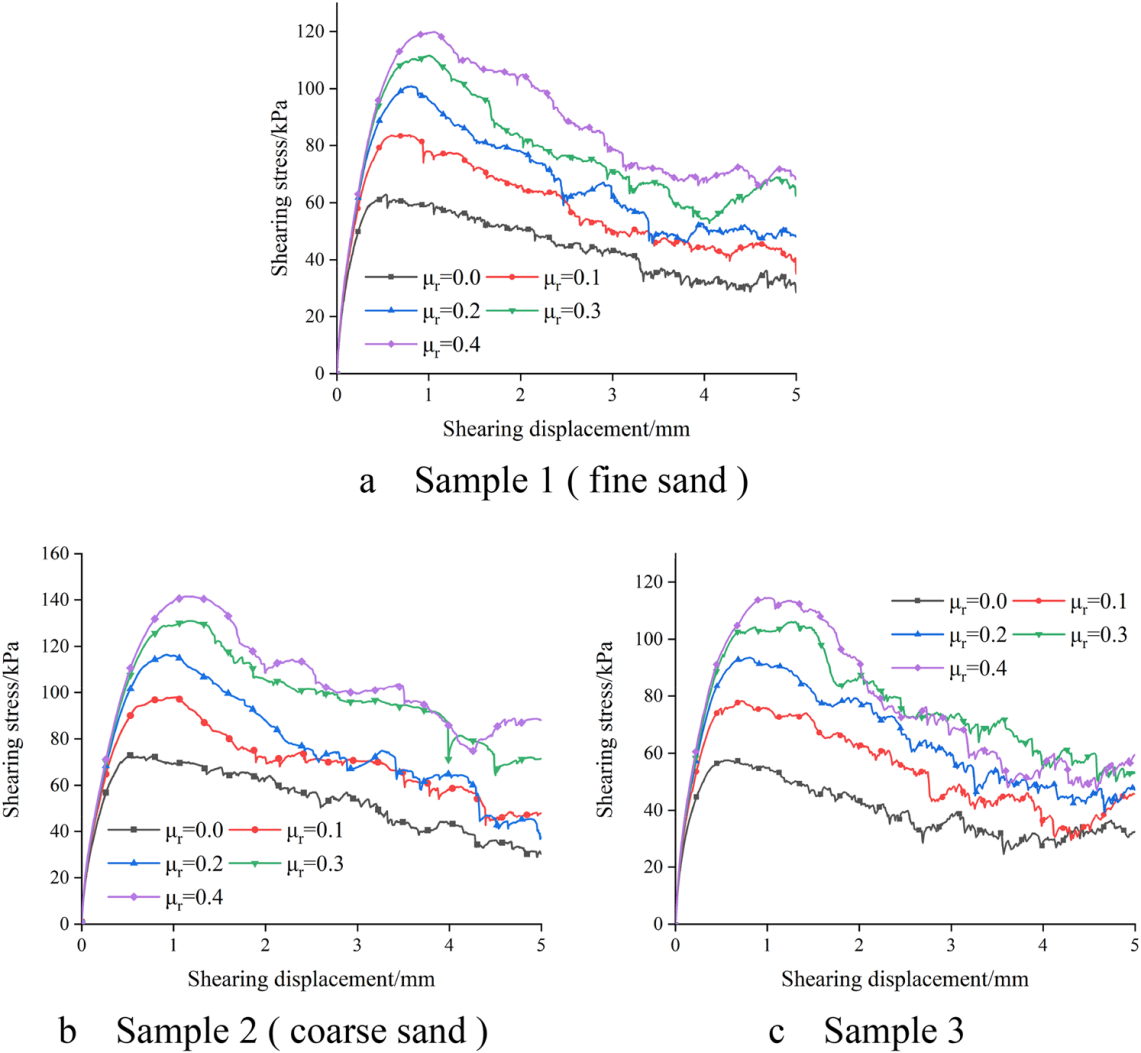
Relation curve of shear stress and shear displacement.

**Figure 5:**
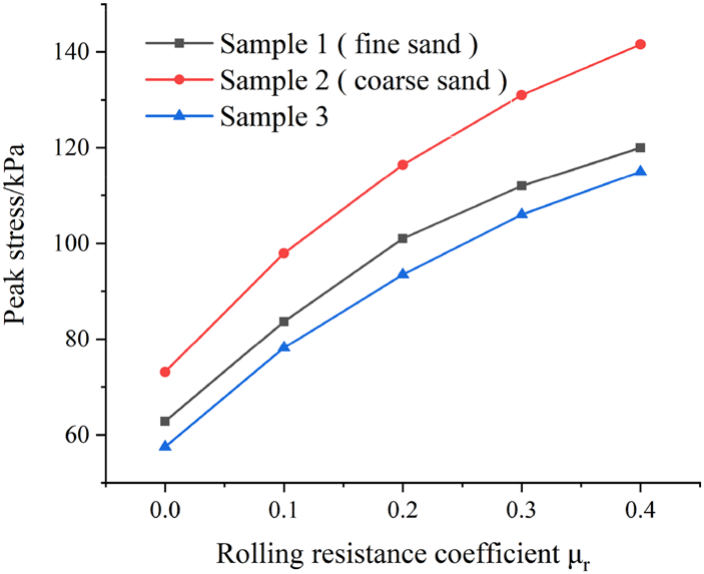
Relation curve between peak stress and anti-rotation coefficient.

**Figure 6:**
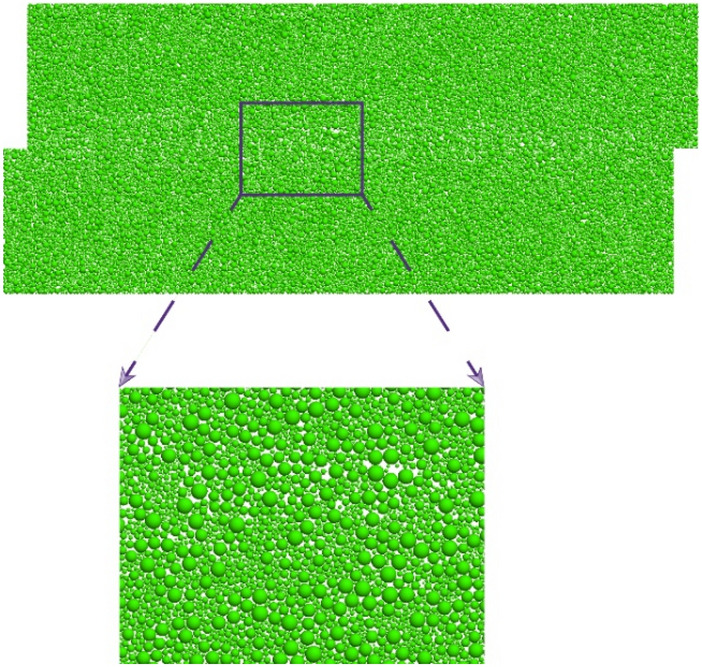
Sample 3 particle distribution.

As shown in Fig. 4, it is the relationship between shear stress and shear displacement under 150 kPa normal stress, different sand samples and different anti-rotation coefficients (Fig. 4). In the early stage of the test shear, in the same sample, the change of the anti-rotation coefficient has little effect on the shear modulus of the sample, and the stress reaches the peak value at a small displacement. With the increase of the anti-rotation coefficient μ_r_, the shear peak stress of the sample increases, and the shear displacement required to reach the peak stress increases. After each sample reaches the peak stress, the stress decreases. The larger the anti-rotation coefficient μ_r_, the more obvious the strain softening phenomenon of the sample. Figure 5 shows the relationship between peak stress and anti-rotation coefficient of different samples (Fig. 5). It can be seen from Fig. 5 that with the increase of anti-rotation coefficient, the effect of anti-rotation coefficient on the growth of peak stress decreases. Under the same conditions, the peak stress of coarse sand is greater than that of fine sand, while the peak stress of sample 3 is the smallest. This is because the larger the particle size, the greater the contact area between particles, the greater the force between particles can withstand, which makes the shear strength of the sample increases. Compared with fine sand, sample 3 contains coarse particles, but the content of coarse particles is less. Coarse particles are sparsely distributed in the sample and cannot form a sand skeleton in the sand sample, which reduces the shear strength of the sample (Fig. 6).

### Dilatancy

**Figure 7:**
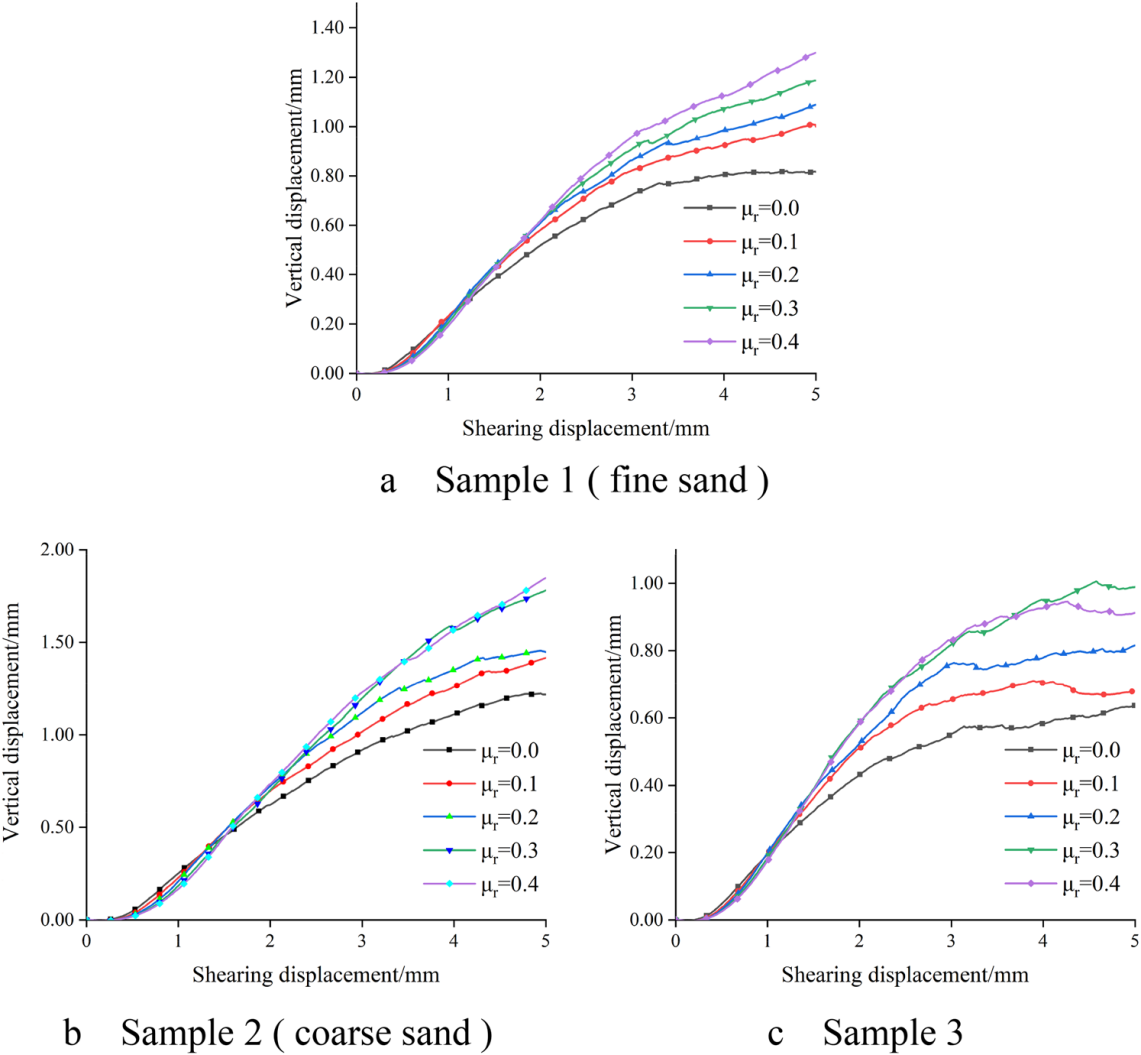
Vertical displacement curves of different samples.

**Figure 8:**
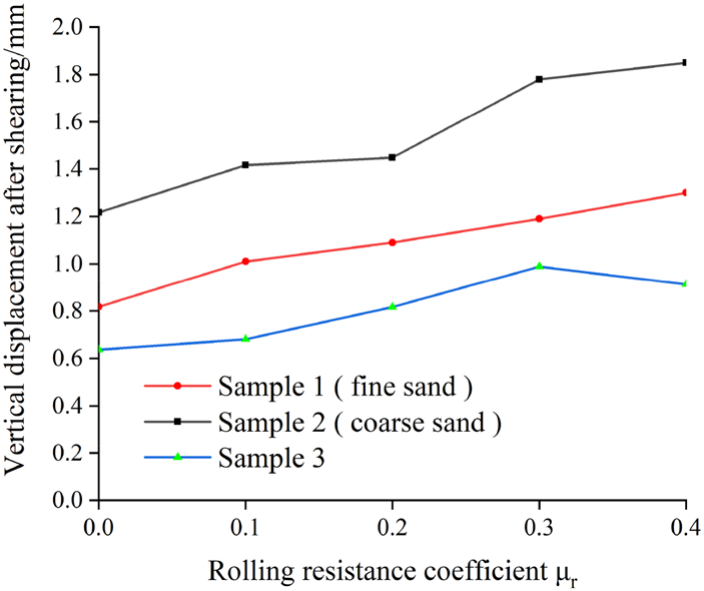
Relation curve between anti-rotation coefficient and vertical displacement after shearing.

Sand dilatancy is mainly controlled by the movement inside the particles during the shear process. The numerical test can well monitor the vertical displacement change of the sample, so as to make up for the deficiency that the indoor experiment cannot accurately monitor the vertical displacement change of the sample. As shown in Fig. 7, the vertical displacement curves of different samples with the change of anti-rotation coefficient under the normal stress of 150 kPa are shown. At the beginning, the three specimens show shear contraction, then begin to show shear dilation. Moreover, as the shear displacement increases, the vertical displacement of the specimen increases. The larger the anti-rotation coefficient, the larger the vertical displacement of the sample, and the larger the corresponding shear displacement when the vertical displacement reaches stability. This is because the greater the anti-rotation ability of the particles, the greater the force required for rotation between the particles, and the increase in the number of particles with dislocation, which shows the more obvious dilatancy of sand. Figure 8 is the relationship curve between the anti-rotation coefficient and the vertical displacement after shearing. It can be seen that the dilatancy phenomenon is more obvious with the increase of the anti-rotation coefficient. The larger the particle size, the smaller the maximum minimum particle size ratio, the more obvious the dilatancy, the greater the vertical displacement.

## Meso-mechanism analysis

### Coordination number change

The coordination number is the number of contacts between the sand particles and the surrounding adjacent particles. The coordination number can be used to describe the tightness of the contact between the particles, which can reflect the compactness and stress level of the sample to a certain extent. The coordination number of the sample is generally expressed by the average number of contacts between particles, the expression is:

4 $$ z = \frac{{2N_{c} }}{{N_{p} }} $$

In the formula: $N_{c}$ is the sum of the number of contacts between particles in the sample; $N_{p}$ is the number of particles in the sample.

**Figure 9:**
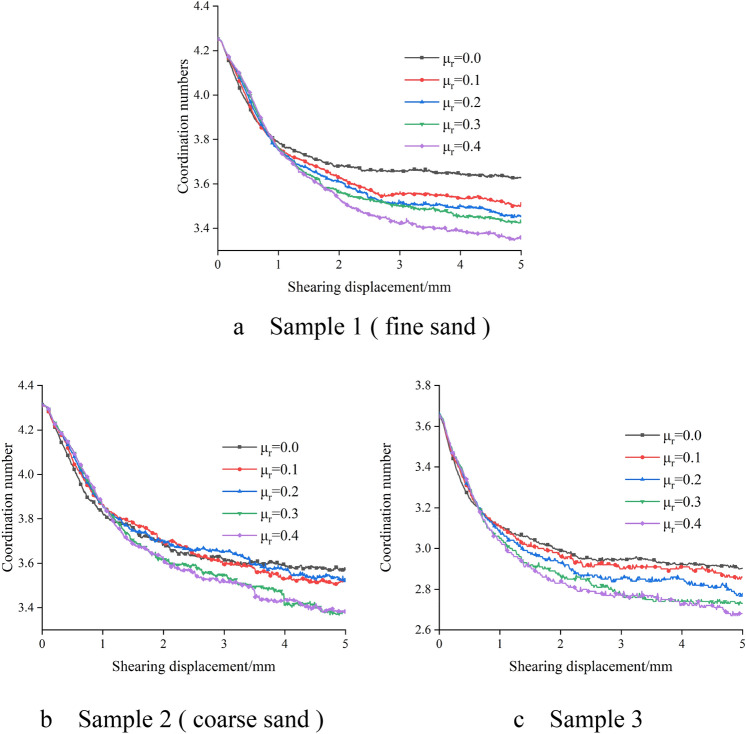
The change curve of coordination number of different samples.

**Figure 10:**
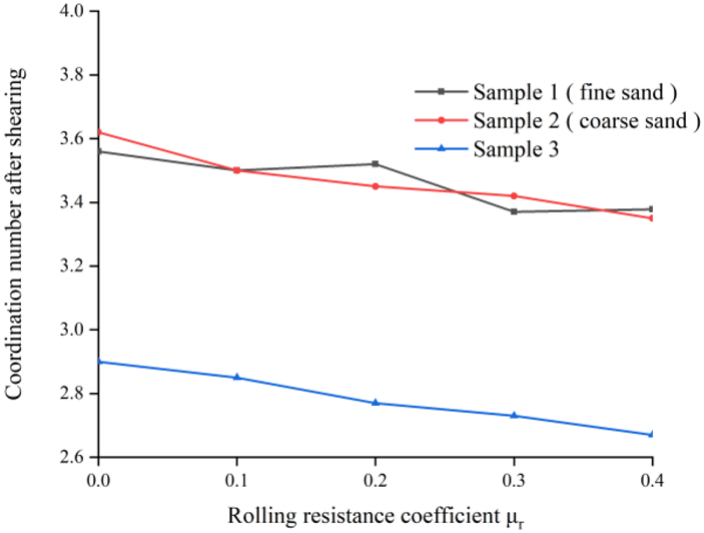
Relation curve between antirotation coefficient and coordination number after shearing.

As shown in Fig. 9, it is the change of coordination number with the change of anti-rotation coefficient in different samples when the normal stress is 150 kPa. It can be seen that the coordination number of the sample decreases rapidly during the shearing process, and then tends to a relatively stable value that no longer changes. When the shear displacement is greater than 1 mm, the change of coordination number slows down, which is similar to the shear displacement when the specimen reaches the peak stress position. The initial coordination number of fine sand and coarse sand is basically the same, while the initial coordination number of sample 3 is lower than that of fine sand and coarse sand. This is because the maximum and minimum particle size ratio of fine sand and coarse sand is 2, while the particle size of sample 3 spans a large range, the maximum and minimum particle size ratio is 7.5, and the contact between particles is not close enough, resulting in the initial coordination number of sample 3 is not high. Figure 10 shows the relationship between the anti-rotation coefficient and the coordination number after shearing. The coordination number after shearing decreases with the increase of the anti-rotation coefficient. The coordination number of coarse sand and fine sand samples changes similarly, while the coordination number of sample 3 is smaller than that of fine sand and coarse sand. This is because the greater the anti-rotation coefficient of sand, the stronger the anti-rotation ability of sand particles, the less likely the relative rotation between particles, the greater the internal friction angle of sand, which in turn makes the formation of greater contact force between particles, the stronger the transfer capacity of force between each particle, the average number of contacts between particles, that is, the coordination number is less.

## Force chain network

**Figure 11:**
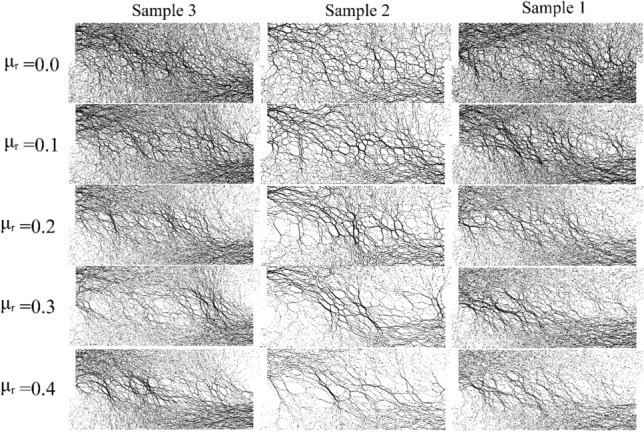
Force chain network of sand after shearing.

The force chain network can reflect the sensitivity of the mechanical response mechanism of the granular material system under the action of external force, which is the macroscopic manifestation of the contact force between particles. As shown in Fig. 11, it is the force chain network after sand shearing. The thicker the contact force chain is, the greater the contact force between particles is. It can be seen that the force chain network distribution of coarse sand is more sparse than that of fine sand and sample 3 under the same conditions, and the force chain skeleton is also larger than that of fine sand and sample 3. This is because in the same volume, the larger the particle size, the less the particle content, the less the number of contacts between particles, and thus the greater the stress borne by each particle, the thicker the force chain. As shown in the figure, as the anti-rotation coefficient increases, the force chain network becomes sparser, the force chain skeleton becomes thicker, the force chain is closer to the shear band, and the force chain exhibits a left dense and right sparse phenomenon, showing the anisotropy of the force chain. With the increase of the anti-rotation coefficient, the strong chain is clearer, the strong chain is more concentrated, and is distributed diagonally along the sample.

## Fabric analysis

**Figure 12:**
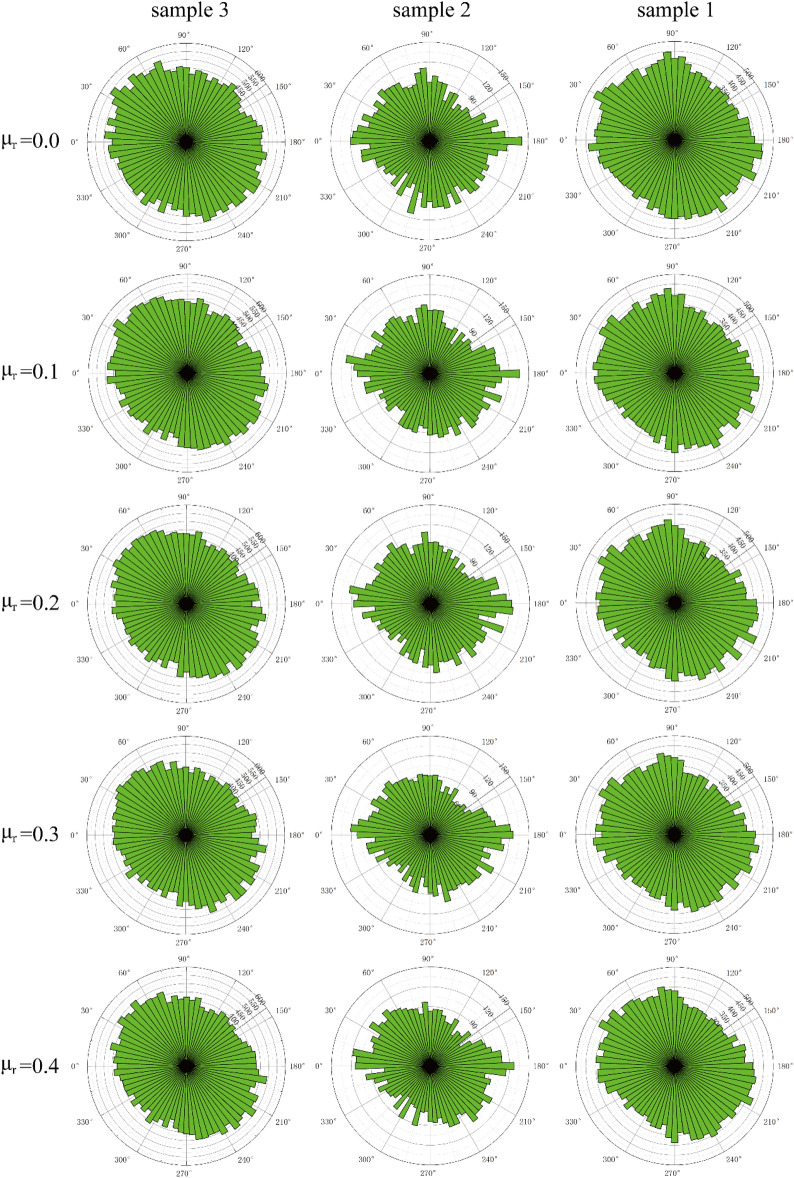
Normal contact force distribution.

In granular materials, contact fabric is used to represent the number distribution map of normal contact direction between particles, which can be used as a common index to reflect the anisotropy of sand samples. As shown in Fig. 12, the distribution of the contact normal fabric in the sample after the shear is completed. It can be seen that in each sample, the number of contacts between particles decreases with the increase of the anti-rotation coefficient, and the proportion of the number of contacts in the 100°–160° direction to the total number of contacts decreases with the increase of the anti-rotation coefficient, and the elliptical shape of the fabric becomes more flat. This is because the anti-rotation coefficient increases, the force to overcome the rotation between particles increases, and then the rotation between particles is not easy to occur, making the dilatancy of the sample more obvious. The porosity becomes larger, so that the contact number of particles becomes less, the force chain from the upper left to the lower right is more concentrated, and the force chain from the lower left to the upper right is more sparse, so that the proportion of the contact number in this direction to the total contact number decreases.

As can be seen from the figure, the area of the pattern increases with the increase of the number of particles in the three samples (Fig. 12). Compared with sample 1 (fine sand) and sample 3, the angle between the principal axes of the fabric in sample 2 (coarse sand) is smaller and the proportion of contact number is larger. This is because the larger the particle size, the smaller the number of particles and the smaller the specific surface area under the same volume. Therefore, the smaller the total number of contacts between particles, the greater the force borne by a single particle, the greater the normal force between particles, the greater the anti-rotation torque between particles, the less likely it is to rotate between particles, and the smaller the angle between the principal axes of contact.

## Porosity analysis

**Figure 13:**
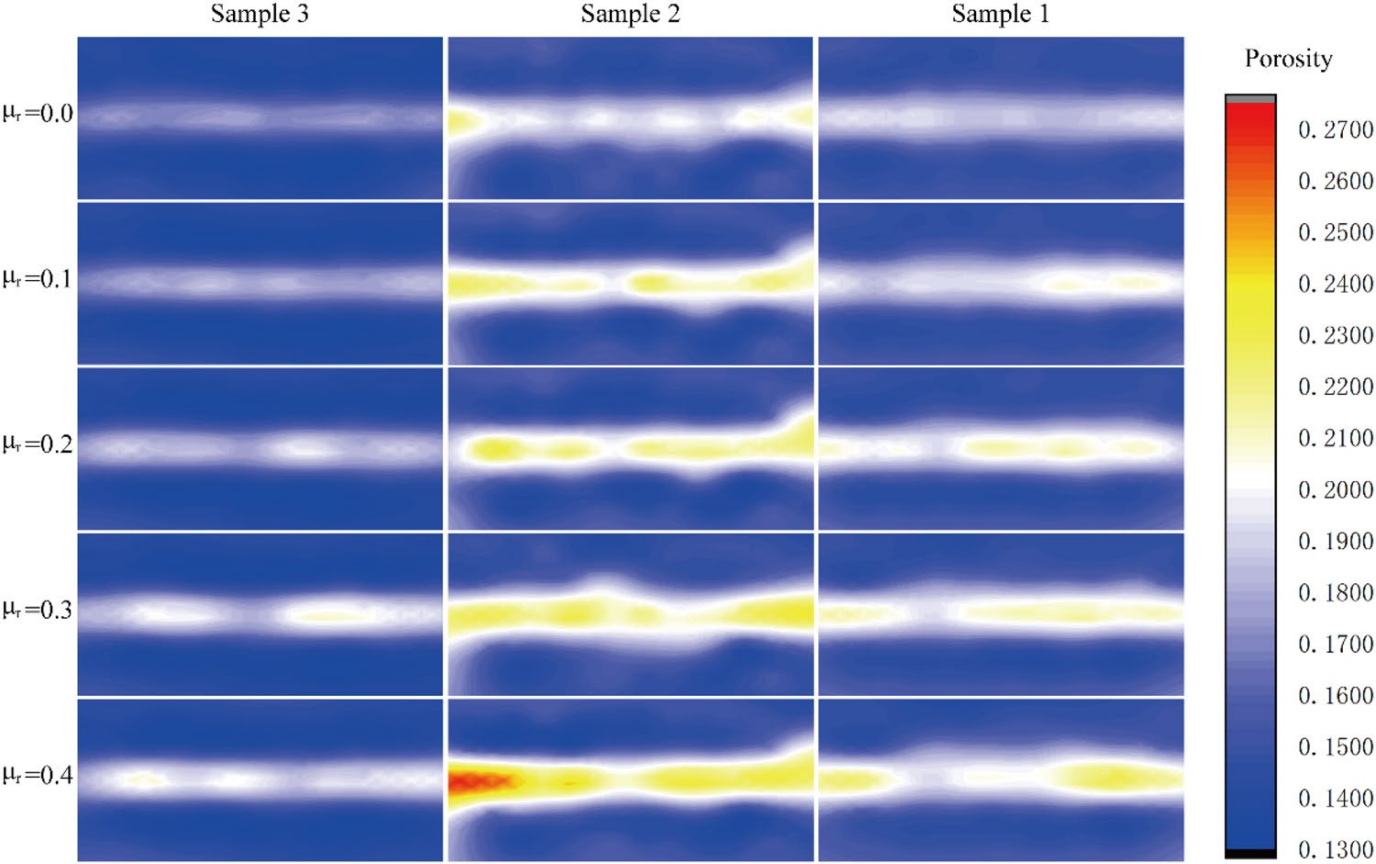
Porosity distribution of sample.

Figure 13 shows the porosity distribution of each group of samples after shearing under different anti-rotation coefficients. It can be seen that the porosity in the middle of the sample increases significantly after shearing, and the rest of the sample changes little. Compared with sample 1 (fine sand) and sample 3, the porosity in the middle of coarse sand changes more greatly in sample 2 (coarse sand). The area where the porosity of the sample changes significantly coincides with the position of the shear band. From the above analysis, it can be seen that the shear stress and the vertical displacement of the sample increase with the increase of the anti-rotation coefficient, which also makes the volume of the sample larger and the porosity of the sample larger, which reflects the dilatancy of sand during shearing. During the shearing process, the shear band is in the middle of the sample, and the dislocation between the particles in other parts is small, so the porosity in the middle of the sample increases obviously.

## Conclusion

The shear properties of sand are closely related to the shape of sand particles. The shape of sand particles is very random, and the numerical test is difficult to reproduce the shape of sand. In this paper, the anti-rotation model is used to simulate the direct shear test of sand to explore the shear characteristics of sand under different anti-rotation coefficients and different sand samples. The following conclusions were reached:

1. The stronger the anti-rotation ability of sand is, the greater the anti-shear strength is. But with the increase of anti-rotation coefficient, the increasing effect of shear strength is weakened, and the strain softening of sand is more obvious after the peak stress is reached.
2. The greater the anti-rotation coefficient, the greater the moment of overcoming relative rotation between particles, the more obvious the dilatancy of the sample, and the more obvious the decrease of the coordination number. The mechanical properties of different samples are quite different. The shear strength and dilatancy of coarse particles are greater than those of fine particles. The maximum and minimum particle size ratio becomes larger, which will weaken the shear strength and dilatancy of the particles.
3. The anti-rotation ability of particles has a great influence on the contact force chain, fabric distribution and porosity change of sand samples. Anti-rotation coefficient increases, the contact strength chain close to the shear band, the more obvious the phenomenon of left and right sparse force chain, the more obvious anisotropy. The ratio of the contact number in the direction of 100°–160° to the total contact number decreases with the increase of the anti-rotation coefficient, the shape of the fabric becomes flatter, and the porosity of the shear band increases obviously.

## Data Availability

The datasets used and/or analysed during the current study available from the corresponding author on reasonable request.
